# Risk factors affecting outcome of rhino-orbital-cerebral mucormycosis in COVID-19 patients

**DOI:** 10.1186/s43163-023-00406-2

**Published:** 2023-06-07

**Authors:** Mina Fayez Saleeb, Sabry Magdy Sabry, Mohammad Salah Mahmoud, Mena Maher Nassif

**Affiliations:** Department of Otolaryngology, Faculty of Medicine, Ain Shams University, Cairo, Egypt

**Keywords:** Rhino-orbito-cerebral mucormycosis, COVID-19, Uncontrolled diabetes mellitus, Invasive fungal infection, Steroids

## Abstract

**Background:**

Mucormycosis is a serious life-threatening fungal infection that recently made severe sudden and devastating surge during the second wave of the COVID-19 epidemic with a mortality rate of up to 50%.

Although the causality link between COVID-19 and rhino-orbito-cerebral mucormycosis (ROCM) remains unclear, many factors including poor diabetes control, high doses of steroids, viral-induced lymphopenia, and cytokine storm have been attributed to ROCM in patients with COVID-19.

Orienting to risk factors and early recognition of this potentially fatal opportunistic infection is the key to optimal management and improved outcomes. In these contexts, we conducted a prospective study for 33 patients admitted to our tertiary hospital to determine the risk factors for ROCM in patients with COVID-19 and the cumulative mortality rates.

**Results:**

This study found a statistically significant relation between the fate of death in COVID-MUCOR patients who had presented fever, ophthalmoplegia, facial skin necrosis, and visual loss with those who received dose of steroid to control their respiratory symptoms *P* < 0.001.

Death from COVID-MUCOR was statistically significant related to the prolonged interval from the onset of the symptoms to start of treatment and intervention. Also, it was found that there was a significant decrease in duration between COVID-19 infection and the start of mucormycosis (days) with incidence of DKA on admission. Nineteen (57.6%) of the patients had uncontrolled diabetes mellitus (hemoglobin A1C (HbA1c) of > 7.0%).

**Conclusion:**

Mucormycosis epidemic was precipitated by a unique confluence of risk factors: diabetes mellitus, widespread use of steroids, and perhaps SARS-CoV-2 infection itself. Restricting steroid use in patients with severe COVID-19 requiring oxygen therapy, and screening for and optimally controlling hyperglycemia, can prevent COVID-MUCOR in a large majority.

## Background

The 2019 coronavirus disease pandemic (COVID-19) has a catastrophic impact throughout the world and has overwhelmed healthcare facilities and caused dramatic increases in excess mortality. Secondary bacterial and fungal infections in patients hospitalized with COVID-19 pneumonia contribute to increased morbidity and mortality. Opportunistic infections, such as mucormycosis, in patients with coronavirus disease 2019 (COVID-19) have become a new health challenge [1].

The surge in COVID-19 cases recorded in Egypt has been associated with an unexpected increase in mucormycosis cases reported in the context of the COVID-19 disease. It has been estimated to be 2.1 times greater in cases in India compared to the pre-pandemic period [2].

The clinical course of mucormycosis is unpredictable, and treatment is challenging and often requires surgery and prolonged antifungal therapy. Mucormycosis is often associated with high mortality even with standard care (approximately 50% and > 90% with disseminated disease) due to the angioinvasive nature of infection that can cause tissue necrosis due to thrombosis and infection dissemination [3].

Since opportunistic infections can exacerbate the status of COVID-19 patients, it is important to identify risk factors to prevent, diagnose, and treat them as soon as possible. Viral, fungal, environmental, and host factors may be responsible for this situation. Long hospital stays, impaired host immune system function due to viral infection, and excessive glucocorticoids consumption in the management of patients with COVID-19 are the main risk factors for the increased risk of mucormycosis in patients with COVID-19 [4].

Other risk factors include comorbidities such as diabetes mellitus, organ transplantation, malignancies (especially hematological), immunosuppressive therapy including corticosteroids, prolonged neutropenia, iron overload, chronic antibiotic use, severe burns, intravenous drug abuse, and malnutrition, leading to immunocompromise [5].

### Aim of work

The purpose of this study is to identify risk factors for the incidence of mucormycosis in COVID-19 patients and correlate the extent of the disease, the condition of the patients, and the intervention plan with the final outcome of the cases.

## Methods

A prospective study involved all patients with mucormycosis presented to the ENT department, Ain Shams University Hospital (Demerdash and Obour Hospitals), in the duration from January 2021 to January 2022.

This study was approved by Ain Shams University Faculty of Medicine Research Ethics Committee (REC) FWA 00017585. Informed written consent to participate in the study was provided by all.

- Detailed history taking of the disease, possible risk factors, and cognitive status
- Full examination including nasal, oral, orbital, and neurological examination
- CT paranasal to assess extension of the disease
- COVID-19 PCR by nasopharyngeal swab to confirm the diagnosis of COVID-19
- Full laboratory investigations
- Management of each case individually as follows: either use of antifungal or surgical debridement with or without exenteration or combination of both
- Statistical analysis was performed for all recorded data. Categorical variables are presented as numbers and percentages, and differences between groups are compared using the Pearson chi-square test or Fisher’s exact test.
- Time-to-event analysis is done using the Kaplan-Meier method.

## Results

**Fig. 1:**
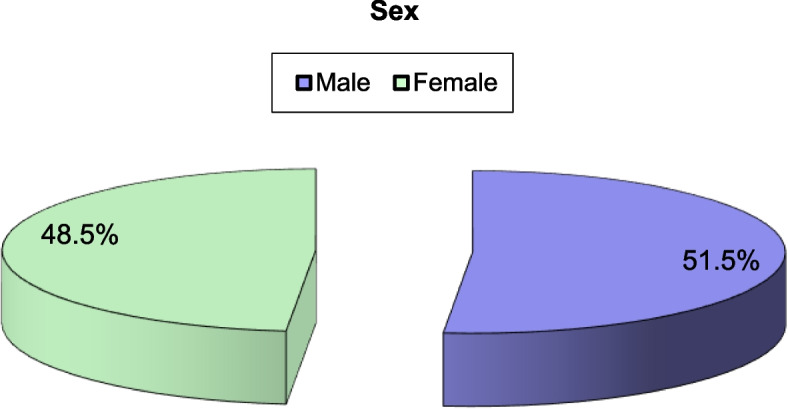
Percentage of female and male patients included in the study

**Fig. 2:**
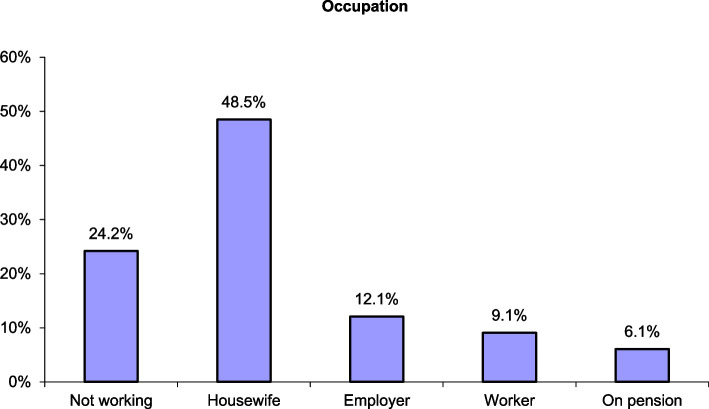
Distribution of occupations of patients included in the study

The study enrolled 33 patients with COVID and mucormycosis (CO-MUCOR). Although the median age of the entire series was 54 years, the ages of the cases ranged widely from 32 to 82 years in the entire analyzed group, with a slight male predominance (51.5% were men, and 48.5% were women), and all the women were housewives. Thirty (90.9%) of the patients were diabetic, either previously known (28 patients) or newly diagnosed (2 patients) (Figs. 1 & 2).

The demographic characteristics of the patients are shown in Table 1. Fasting blood sugar was markedly elevated, and about 26.7% of the patients had coexisting diabetic ketoacidosis on admission. Details are presented in the documentation (Table 1).

**Table 1:** The demographic characteristics of the patients

	Total no. = 33
**Age**	
Mean ± SD	54.27 ± 12.73
Range	32–82
**Sex**	
Male	17 (51.5%)
Female	16 (48.5%)
**Occupation**	
Not working	8 (24.2%)
Housewife	16 (48.5%)
Employer	4 (12.1%)
Worker	3 (9.1%)
On pension	2 (6.1%)
**Smoking**	8 (24.2%)
**Diabetes**	30 (90.9%)
**Controlled**	11 (36.7%)
**Uncontrolled**	19 (57.6%)
**Newly diagnosed**	2 (6.7%)
**DKA on admission**	8 (26.7%)
**Immunosuppressive drugs**	0 (0.0%)
**Chronic liver disease**	1 (3.0%)
**Chronic kidney disease**	1 (3.0%)
**Hematologic malignancy**	0 (0.0%)
**Prolonged antibiotic or antiviral therapy**	9 (27.3%)
**Autoimmune disease**	1 (3.0%)
**Prolonged steroid use**	29 (88%)
**Chemotherapy**	0 (0.0%)
**Thrombocytopenia**	2 (6.1%)

Nineteen (57.6%) of the patients had uncontrolled diabetes mellitus (hemoglobin A1C (HbA1c) of > 7.0%).

Regarding symptoms, most of patients (69.7%) with COVID-MUCOR had headache, facial pain, and orbital swelling. About half of the patients firstly presented with eye swelling (55.5%) or had ophthalmoplegia (54.5%) and visual loss (48.5%) (Table 2).

**Table 2:** Symptomatology at presentation

	Total no. = 33
**Fever**	14 (42.4%)
**Facial numbness**	20 (60.6%)
**Facial swelling**	19 (57.6%)
**Headache**	23 (69.7%)
**Facial pain**	23 (69.7%)
**Rhinorrhea, blood stained**	11 (33.3%)
**Orbital swelling**	23 (69.7%)
**Ophthalmoplegia**	18 (54.5%)
**Visual loss**	16 (48.5%)
**Disturbed conscious**	8 (24.2%)
**Motor or sensory weakness (stroke)**	6 (18.2%)
**First presentation**	
Eye swelling	5 (55.6%)
Disturbed conscious	3 (33.3%)
Facial swelling	1 (11.1%)
**Intranasal necrosis**	29 (87.9%)
**Palatal necrosis**	19 (57.6%)
**Ophthalmoplegia**	18 (54.5%)
**Proptosis**	13 (39.4%)
**Visual loss**	16 (48.5%)
**Cavernous sinus thrombosis**	18 (54.5%)
**Facial skin necrosis**	2 (6.1%)
**Cranial nerves involvement**	
Negative	27 (81.8%)
Facial	6 (18.2%)

On examination, most patients (87.9%) with COVID-MUCOR had intranasal necrosis, and more than half (57.6%) had palatal necrosis (Table 2).

A total of 66.7% of CO-MUCOR patients had previously known COVID, and the median CT CO-RAD score in patients with COVID-MUCOR in this study was 3, and the median duration between COVID-19 infection and the start of mucormycosis was 30 days (Table 3).

**Table 3:** Radiological score

	Total no. = 33
**CT PNS**	
Rt max. S	16 (48.5%)
Lt max. S	26 (78.8%)
Rt ethmoid	20 (60.6%)
Lt ethmoids	27 (81.8%)
Rt sphenoid	19 (57.6%)
Lt sphenoid	24 (72.7%)
Rt frontal	13 (39.4%)
Lt frontal	15 (45.5%)
Bony erosions	17 (51.5%)
Bilateral involvement	14 (42.4%)
**COVID status**	
Previous COVID	22 (66.7%)
PCR at presentation	6 (18.2%)
**CT CO-RAD score**	
Median (IQR)	3 (1–4)
Range	1–5

Steroid use was common, even in mild disease, and was strongly associated with COVID-MUCOR (*P* < 0.001). In the majority (88%, 29 patients) of the study, patients had received high-dose steroids (> 40 mg of prednisolone or equivalent) for the management of COVID-19. Only 18.2% had received anti-IL-6 actemra (tocilizumab) or antiviral (remdesivir) (Table 4).

**Table 4:** Previous medication during COVID treatment period

Duration between COVID-19 infection and start of mucormycosis	
Median (IQR)	30 (21–60)
Range	7–90
**Steroids dose**	29 (88%)
**Anti-IL-6 actemra (tocilizumab)**	6 (18.2%)
**Antiviral (remdesivir)**	6 (18.2%)

Regarding sinus affection in CT scan, pansinusitis was the most common involvement followed by the left ethmoid sinus (27 patients, 81.8%), followed by the left maxillary sinus (26 patients, 78.8), and followed by the Lt sphenoid sinus (24 patients, 72.7%). A total of 57.6% of patients with CO-MUCOR had unilateral sinus affection (Table 3).

In our study, all patients received systemic antifungal therapy (amphoterecin B). About two-thirds of the patients (24 patients, 72.7%) underwent endoscopic sinus surgery for mucormycosis, 7 patients (21.2%) underwent combined (endoscopic & external) management, and only 2 patients (6.1%) were managed by external approach to the paranasal sinuses.

Repeated surgical intervention was needed in some cases, 8 patients (25.0%) underwent two surgeries, and 1 (3.6%) patient underwent 3 surgical interventions. During sinus surgery (ESS), most of included patients needed inferior turbinectomy (97.0%), middle turbinectomy (93.9%), middle meatal antrostomy (97.0%), and ethmoidectomy (93.9%). Nine of the patients had died during the study period (Table 5).

**Table 5:** Extent of intervention done and fate

	Total no. = 33
**Interval from symptom onset to treatment**	
Median (IQR)	7 (4–10)
Range	1–63
**Endoscopic treatment**	24 (72.7%)
**Combined (endoscopic and external)**	7 (21.2%)
**External**	2 (6.1%)
**Total no. of surgical procedures**	
0	2 (7.1%)
1	18 (64.3%)
2	7 (25.0%)
3	1 (3.6%)
**Inf. turb.**	32 (97.0%)
**Middle turb.**	31 (93.9%)
**MMA**	32 (97.0%)
**Septectomy**	17 (51.5%)
**Palatal debrid.**	3 (9.1%)
**Ethmoidectomy**	31 (93.9%)
**Sphenoidotomy**	25 (75.8%)
**Death**	9 (27.3%)
**Onset of death or discharge (days)**	
Median (IQR)	30 (14–30)
Range	5–90

In the current study, we found that there was a significant decrease in duration between COVID-19 infection and the start of mucormycosis (days) with incidence of DKA on admission with median (IQR) = (14 (14–23) vs. 40 (25–75)) and *P*-value = 0.018.

In addition, a significant decrease in duration between −19 infection and the start of mucormycosis was observed in patients with facial swelling (*P*-value 0.043) and proptosis (*P*-value 0.046) (Tables 5 & 6) and in those who received prolonged antibiotic or antiviral therapy (*P*-value 0.011) (Tables 6, 7, & 8).

**Table 6:** Duration between COVID-19 infection and start of mucormycosis

	Duration between COVID-19 infection and start of mucormycosis (days)		Test value	***p***-value	Sig.
	Median (IQR)	Range			
Sex					
Male	30 (21–60)	14–90	−0.298^a^	0.765	NS
Female	30 (20–45)	7–90			
Occupation					
Not working	30 (25–40)	21– 0	1.124^b^	0.891	NS
Housewife	30 (20–45)	7–90			
Employer	21 (14–90)	14–90			
Worker	37 (14–60)	14–60			
On pension	60 (60–60)	60–60			
Smoking					
No	30 (20–60)	7–90	−0.213^a^	0.832	NS
Yes	25 (21–60)	14–80			
Diabetes					
No	25.5 (21–30)	21–30	−0.517^a^	0.605	NS
Yes	30 (20.5–60)	7–90			
Controlled					
No	40 (14–60)	7–80	−0.305^a^	0.760	NS
Yes	30 (21–60)	14–90			
Newly diagnosed					
No	30 (21–60)	7–90	−0.958^a^	0.338	NS
Yes	20 (20–20)	20–20			
DKA on admission					
No	40 (25–75)	14–90	−2.367^a^	**0.018**	S
Yes	14 (14–20)	7–45			
Prolonged antibiotic or antiviral therapy					
No	42.5 (30–60)	14–90	−2.540^a^	**0.011**	S
Yes	20.5 (14–23)	7–80			
Prolonged steroid use					
No	30 (21–60)	7–90	−0.714^a^	0.476	NS
Yes	21 (21–21)	21–21			
Fever					
No	30 (21–67.5)	7–90	−1.001^a^	0.317	NS
Yes	25 (14–45)	14–60			
Facial numbness					
No	27.5 (21–30)	14–90	−0.398^a^	0.691	NS
Yes	42.5 (17–60)	7–90			
Facial swelling					
No	37.5 (27.5–67.5)	21–90	−2.023^a^	**0.043**	S
Yes	20.5 (14–40)	7–90			
Headache					
No	30 (25–80)	21–90	−0.993^a^	0.321	NS
Yes	30 (14–60)	7–90			
Facial pain					
No	30 (25–80)	21–90	−0.853^a^	0.394	NS
Yes	30 (17–52.5)	7–90			
Rhinorrhea, blood stained					
No	21 (14–75)	7–90	−1.418^a^	0.156	NS
Yes	45 (30–60)	25–60			
Orbital swelling					
No	50 (25.5–85)	14–90	−1.648^a^	0.099	NS
Yes	27.5 (20–45)	7–75			
Ophthalmoplegia					
No	30 (21–60)	7–90	−0.429^a^	0.668	NS
Yes	30 (20–60)	14–80			

*P*-value > 0.05, nonsignificant; *P*-value < 0.05, significant; *P*-value < 0.01, highly significant

The previous table shows that there was significant decrease in duration (days) with incidence of DKA on admission with median (IQR) = (14 (14–23) vs 40 (25–75)) and *P*-value = 0.018

^a^Mann-Whitney test

^b^Kruskal-Wallis test

**Table 7:** Duration between covidCOVID-19 infection and start of mucormycosis

	Duration between COVID-19 infection and start of mucormycosis		Test value	***p***-value	Sig.
	Median (IQR)	Range			
Visual loss					
No	30 (21–45)	7–90	−0.134^a^	0.893	NS
Yes	30 (21–60)	14–80			
Disturbed conscious					
No	30 (25–60)	7–90	−1.892^a^	0.059	NS
Yes	20 (14–21)	14–45			
Motor or sensory weakness (stroke)					
No	30 (25–60)	7–90	−1.222^a^	0.222	NS
Yes	21 (20–21)	14–80			
First presentation					
Eye swelling	27.5 (23–30)	21–30	4.909^b^	0.086	NS
Disturbed conscious	17 (14–20)	14–20			
Facial swelling	40 (40–40)	40–40			
Intranasal necrosis					
No	20 (20–20)	20–20	−1.031^a^	0.303	NS
Yes	30 (21–60)	7–90			
Palatal necrosis					
No	37.5 (25–82.5)	14–90	−1.133^a^	0.257	NS
Yes	27.5 (21–45)	7–80			
Ophthalmoplegia					
No	30 (20–60)	7–90	−0.165^a^	0.869	NS
Yes	30 (21–60)	14–80			
Proptosis					
No	42.5 (30–75)	7–90	−1.991^a^	**0.046**	S
Yes	21 (17–27.5)	14–60			
Visual loss					
No	30 (21–45)	7–90	−0.134^a^	0.893	NS
Yes	30 (21–60)	14–80			
Cavernous sinus thrombosis					
No	30 (25–60)	14–90	−1.123^a^	0.261	NS
Yes	21 (14–60)	7–80			
Facial skin necrosis					
No	30 (21–60)	7–90	−1.348^a^	0.178	NS
Yes	14 (14–14)	14–14			
Cranial nerves involvement					
Negative	30 (21–45)	14–90	−0.128^a^	0.898	NS
Facial	40.5 (14–67.5)	7–75			

*P*-value > 0.05, nonsignificant; *P*-value < 0.05, significant; *P*-value < 0.01, highly significant

^a^Mann-Whitney test

^b^Kruskal-Wallis test

**Table 8:** Duration between covidCOVID-19 infection and start of mucormycosis

	Duration between COVID-19 infection and start of mucormycosis		Test value	***p***-value	Sig.
	Median (IQR)	Range			
Total no. of surgical procedures					
1	27.5 (17–57.5)	14–90	0.078^b^	0.962	NS
2	25.5 (21–60)	7–90			
3	30 (30–30)	30–30			
Septectomy					
No	23 (14–60)	7–90	−0.995^a^	0.320	NS
Yes	30 (25.5–60)	14–90			
Palatal debrid					
No	30 (20.5–52.5)	7–90	−0.632^a^	0.527	NS
Yes	55.5 (21–90)	21–90			
Sphenoidotomy					
No	14 (10.5–22)	7–30	−2.355^a^	**0.019**	S
Yes	35 (21–60)	14–90			
Death					
No	30 (21–60)	7–90	−0.355^a^	0.723	NS
Yes	30 (14–45)	14–80			

*P*-value > 0.05, nonsignificant; *P*-value < 0.05, significant; *P*-value < 0.01, highly significant

^a^Mann-Whitney test

^b^Kruskal-Wallis test

**Fig. 3:**
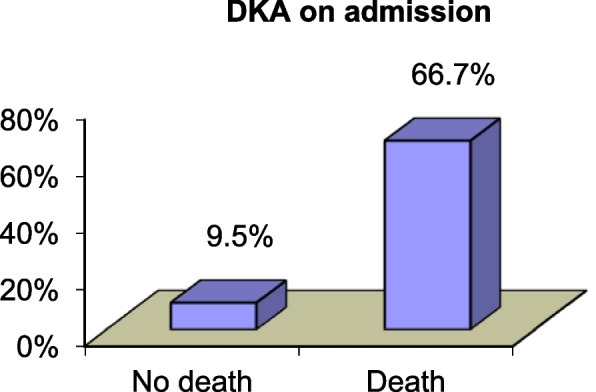
Correlation between DKA and fate of the disease

All patients who died were diabetics, and nearly two-thirds had coexisting ketoacidosis on admission. Therefore, we found a highly statistically significant relation between the presence of diabetic ketoacidosis (DKA) and then aggressiveness of mucormycosis that ends in death in patients with COVID with *P*-value = 0.001 (Table 9, Fig. 3).

**Table 9:** Comparison of demographics between patients who had an adverse outcome (death) vs. those who were alive

	Alive	Death	Test value	***p***-value	Sig.
	No. = 24	No. = 9			
**Age**					
Mean ± SD	54.75 ± 11.05	53.00 ± 17.18	0.347^b^	0.731	NS
Range	32–78	34–82			
**Sex**					
Male	13 (54.2%)	4 (44.4%)	0.248^a^	0.619	NS
Female	11 (45.8%)	5 (55.6%)			
**Occupation**					
Not working	7 (29.2%)	1 (11.1%)	2.855^a^	0.582	NS
Housewife	11 (45.8%)	5 (55.6%)			
Employer	2 (8.3%)	2 (22.2%)			
Worker	2 (8.3%)	1 (11.1%)			
On pension	2 (8.3%)	0 (0.0%)			
**Smoking**	6 (25.0%)	2 (22.2%)	0.028^a^	0.868	NS
**Diabetes**	21 (87.5%)	9 (100.0%)	1.238^a^	0.266	NS
**Controlled**	9 (42.9%)	2 (22.2%)	1.155^a^	0.282	NS
**Newly diagnosed**	1 (4.8%)	1 (11.1%)	0.408^a^	0.523	NS
**DKA on admission**	2 (9.5%)	6 (66.7%)	10.519^a^	**0.001**	HS
**Immunosuppressive drugs**	0 (0.0%)	0 (0.0%)			
**Chronic liver disease**	1 (4.2%)	0 (0.0%)	0.387^a^	0.534	NS
**Chronic kidney disease**	1 (4.2%)	0 (0.0%)	0.387^a^	0.534	NS
**Hematologic malignancy**	0 (0.0%)	0 (0.0%)	–	–	–
**Prolonged antibiotic or antiviral therapy**	5 (20.8%)	4 (44.4%)	1.840^a^	0.175	NS
**Autoimmune disease**	1 (4.2%)	0 (0.0%)	0.387^a^	0.534	NS
**Prolonged steroid use**	29 (88%)	0 (0.0%)	1.238^a^	0.266	NS
**Chemotherapy**	0 (0.0%)	0 (0.0%)	–	–	–
**Thrombocytopenia**	2 (8.3%)	0 (0.0%)	0.798^a^	0.372	NS

*P*-value > 0.05, nonsignificant; *P*-value < 0.05, significant; *P*-value < 0.01, highly significant

^a^Chi-square test

^b^Independent *t*-test

Also, we found a statistically significant increase in the probability of death in those who had received prolonged high steroid use during COVID management (Table 9).

In the present study, all patients who had facial skin necrosis died. And most of patients (8 out of 9) who died had orbital swelling ophthalmoplegia and intranasal necrosis (88.9% for each). Most of the dead (7 of 9) had fever, headache, facial pain, visual loss, and cavernous sinus thrombosis (77.8% for each).

**Fig. 4:**
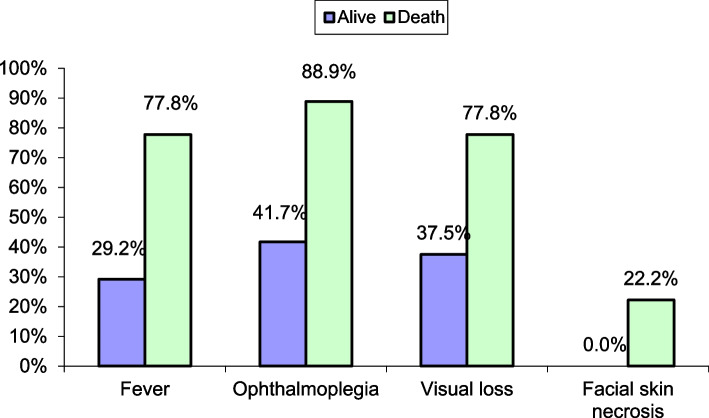
Correlation of symptoms and signs and the fate of the disease

After statistical analysis, we found a statistically significant relation between the fate of death in COVID-MUCOR patients who had presented fever, ophthalmoplegia, facial skin necrosis, and visual loss (with *P*-value of 0.012, 0.015, 0.017, and 0.039, respectively) (Table 10) and with those who received dose of steroid control their respiratory symptoms *P* < 0.001 (Table 11, Fig. 4).

**Table 10:** Comparison of clinical characteristics between patients who died vs. those who were alive

	Alive	Death	Test value	***p***-value	Sig.
	No. = 24	No. = 9			
**Fever**	7 (29.2%)	7 (77.8%)	6.332*	**0.012**	S
**Facial numbness**	15 (62.5%)	5 (55.6%)	0.132*	0.716	NS
**Facial swelling**	13 (54.2%)	6 (66.7%)	0.419*	0.518	NS
**Headache**	16 (66.7%)	7 (77.8%)	0.383*	0.536	NS
**Facial pain**	16 (66.7%)	7 (77.8%)	0.383*	0.536	NS
**Rhinorrhea, blood stained**	7 (29.2%)	4 (44.4%)	0.688*	0.407	NS
**Orbital swelling**	15 (62.5%)	8 (88.9%)	2.158*	0.142	NS
**Ophthalmoplegia**	10 (41.7%)	8 (88.9%)	5.887*	**0.015**	S
**Visual loss**	9 (37.5%)	7 (77.8%)	4.251*	**0.039**	S
**Disturbed conscious**	4 (16.7%)	4 (44.4%)	2.750*	0.097	NS
**Motor or sensory weakness (stroke)**	4 (16.7%)	2 (22.2%)	0.136*	0.712	NS
**First presentation**					
Eye swelling	5 (62.5%)	0 (0.0%)	2.250*	0.325	NS
Disturbed conscious	2 (25.0%)	1 (100.0%)			
Facial swelling	1 (12.5%)	0 (0.0%)			
**Intranasal necrosis**	21 (87.5%)	8 (88.9%)	0.012*	0.913	NS
**Palatal necrosis**	13 (54.2%)	6 (66.7%)	0.419*	0.518	NS
**Proptosis**	8 (33.3%)	5 (55.6%)	1.354*	0.245	NS
**Cavernous sinus thrombosis**	11 (45.8%)	7 (77.8%)	2.694*	0.101	NS
**Facial skin necrosis**	0 (0.0%)	2 (22.2%)	5.677*	**0.017**	S
**Cranial nerves involvement**					
Negative	18 (75.0%)	9 (100.0%)	2.750*	0.097	NS
Facial	6 (25.0%)	0 (0.0%)

**Table 11:** IIncidence of death compared to various factors

	No death	Death	Test value	***p***-value	Sig.
	No. = 24	No. = 9			
**CT PNS**					
**Rt max. S**	12 (50.0%)	4 (44.4%)	0.081*	0.776	NS
**Lt max. S**	18 (75.0%)	8 (88.9%)	0.755*	0.385	NS
**Rt ethmoid**	15 (62.5%)	5 (55.6%)	0.132*	0.716	NS
**Lt ethmoids**	19 (79.2%)	8 (88.9%)	0.416*	0.519	NS
**Rt sphenoid**	14 (58.3%)	5 (55.6%)	0.021*	0.886	NS
**Lt sphenoid**	18 (75.0%)	6 (66.7%)	0.229*	0.632	NS
**Rt frontal**	9 (37.5%)	4 (44.4%)	0.132*	0.716	NS
**Lt frontal**	12 (50.0%)	3 (33.3%)	0.733*	0.392	NS
**Bony erosions**	13 (54.2%)	4 (44.4%)	0.248*	0.619	NS
**Bilateral involvement**	10 (41.7%)	4 (44.4%)	0.021*	0.886	NS
**COVID status**					
**Previous COVID**	17 (70.8%)	5 (55.6%)	0.688*	0.407	NS
**PCR at presentation**	3 (12.5%)	3 (33.3%)	1.910*	0.167	NS
**CT CO-RAD score**					
Median (IQR)	2 (1–3)	3.5 (2.5–4.5)	−1.951≠	0.051	NS
Range	1–5	1–5			
**Duration between COVID-19 infection and start of mucormycosis**					
Median (IQR)	30 (21–60)	30 (14–45)	−0.355≠	0.723	NS
Range	7–90	14–80			
**Steroids use**	20 ( 83%)	9 (100%)	0.001*	0.001	HS
**Anti-IL-6 actemra (tocilizumab)**	4 (16.7%)	2 (22.2%)	0.136*	0.712	NS
**Antiviral (remdesivir)**	4 (16.7%)	2 (22.2%)	0.136*	0.712	NS

Death from COVID-MUCOR was statistically significant related to the prolonged interval from the onset of the symptoms to the start of treatment and intervention (Tables 12 & 13).

**Table 12:** Incidence of death compared to various factors

	No death	Death	Test value	***p***-value	Sig.
	No. = 24	No. = 9			
**Interval from symptom onset to treatment**					
Median (IQR)	8 (5–12)	5 (3–7)	−1.524^b^	0.127	NS
Range	2–63	1–14			
**Endoscopic treatment**	19 (79.2%)	5 (55.6%)	1.840^a^	0.175	NS
**Combined (endoscopic and external)**	4 (16.7%)	3 (33.3%)	1.088^a^	0.297	NS
**External**	2 (8.3%)	0 (0.0%)	0.798^a^	0.372	NS
**Total no. of surgical procedures**					
0	1 (4.8%)	1 (14.3%)	4.000^a^	0.261	NS
1	12 (57.1%)	6 (85.7%)			
2	7 (33.3%)	0 (0.0%)			
3	1 (4.8%)	0 (0.0%)			
**Inf. turb.**	23 (95.8%)	9 (100.0%)	0.387^a^	0.534	NS
**Middle turb.**	22 (91.7%)	9 (100.0%)	0.798^a^	0.372	NS
**MMA**	23 (95.8%)	9 (100.0%)	0.387^a^	0.534	NS
**Septectomy**	11 (45.8%)	6 (66.7%)	1.137^a^	0.286	NS
**Palatal debrid.**	2 (8.3%)	1 (11.1%)	0.061^a^	0.805	NS
**Ethmoidectomy**	22 (91.7%)	9 (100.0%)	0.798^a^	0.372	NS
**Sphenoidotomy**	20 (83.3%)	5 (55.6%)	2.750^a^	0.097	NS

*P*-value > 0.05, nonsignificant; *P*-value < 0.05, significant; *P*-value < 0.01, highly significant

^a^Chi-square test

^b^Mann-Whitney test

•Independent *t*-test

**Table 13:** Relation between interval from symptom onset to treatment and onset of death or discharge

	Interval from symptom onset to treatment	
	***r***	***p***-value
Onset of death or discharge (days)	0.153	0.396

*P*-value > 0.05, nonsignificant; *P*-value < 0.05, significant; *P*-value < 0.01, highly significant. Spearman correlation coefficient

DiscussionMucormycosis is a sporadic disease that occurs almost exclusively in immunosuppressed patients. The sudden spike in the incidence of mucormycosis in the COVID-19 pandemic raises the possibility that COVID-19 infection may itself predispose to mucormycosis. This may occur directly through its impact on the immune system or indirectly due to interventions related to COVID-19 prevention and management [6].

It should be mentioned that our current understanding of the link between COVID-19 and mucormycosis is incomplete, based on observational analyses, and invalidated by basic experimentation. A link between both infections could be through the biochemical alterations caused by the viral infection, and DM and corticosteroid therapy just contribute to such alteration [7].

We report on the experience from Egypt where we studied a total of 33 patients with COVID and mucormycosis (CO-MUCOR) in Ain Shams University Hospitals during the third pandemic wave in the country that spanned the summer of 2021.

Most of the CO-MUCOR cases globally reported so far is from the Indian population. However, the burden of mucormycosis in the Indian population was high before the beginning of the COVID-19 pandemic. The country has also further experienced a surge in COVID-19 cases during the second wave, probably relating to prevalent strain variants [8].

The largest national-wide survey of CO-MUCOR to date is the collaborative OPAI-IJO study on mucormycosis in COVID-19 (COSMIC), by Sen et al. in 2021, which analyzed the data of 2826 patients who presented from the start of the pandemic January 2020 until May 2021. DM was present in 78% of the patients, and 87% had received corticosteroids for COVID-19, suggesting that both factors are the most important predisposing factors for CO-MUCOR. This is in line with the findings of our study, in which 90.9% of the patients had DM and 88% had a history of corticosteroid treatment [9].

The prevalence of diabetes in CO-MUCOR in our study was higher than historical cohorts of mucormycosis (Binder et al., 2014) not associated with COVID (90.9% vs. 76%). Newly detected diabetes is reportedly less prevalent in CO-MUCOR compared to mucormycosis not associated with COVID-19 (6% versus 10%). This disagreed with Arora et al. in 2022 who performed a case-control study of 352 patients (152 cases and 200 controls) diagnosed with COVID-19, and they found that newly detected diabetes was much higher in CO-MUCOR patients (20%) [6, 10].

Diabetic ketoacidosis is detected in 26.7% of patients with mucormycosis at presentation, which is a rare occurrence in the natural history of type 2 diabetes; otherwise [11], this agrees with Arora et al. (2022) who observed the appearance of DKA in 22% of CO-MUCOR at presentation [6]. However, DKA was previously identified to be uncommon among CO-MUCOR compared to mucormycosis not associated with COVID-19 (8.6%) in the study of Patel et al. in 2021 [2].

Reports of CO-MUCOR outside the Indian population are scarce, most of which are case reports (Hussain et al., 2021) [12]. In Iran, two cross-sectional studies in 2021 from Tehran by Pakdel et al. and West Iran by Avatef Fazeli et al. have reported on 15 and 12 cases of CO-MUCOR of which 86% and 87.3% had DM, with a mortality rate of 47% and 66.6%, respectively. These two studies agree with our study in percent of diabetics, but they reported a markedly higher mortality rate that may be due to smaller sample size than our current study [13, 14].

Our study also agrees with a series of 10 cases with CO-MUCOR reported in Pakistan by Nasir et al. in 2021 of which 70% were diabetic and 80% had received corticosteroids [15].

In contrast to our study, Rabagliati et al. in 2021 had reported 16 cases from Chile, 15 of which had received corticosteroids, but only 4 (25%) were diabetic, and none was immunocompromised, suggesting different predisposing factors within different settings [16].

In Egypt, Fouad et al., in December 2021, performed a multicentric retrospective analysis of 26 patients with CO-MUCOR, 96.2% had poorly controlled diabetes mellitus, and 76.9% had received corticosteroid treatment for COVID-19. They also reported that 61.6% of CO-MUCOR patients had visual loss. These findings come in accordance of our study (90.9% DM, 88% STEROID, 48.5% presented with visual loss). But on the other hand, Fouad et al. reported a shorter median duration from COVID-19 until the onset of CO-MUCOR was 20.5 days versus 30 days in our study, and a higher mortality rate was 46.2% despite surgical debridement in half of the cases that died (27.3% in our study) [17].

In older studies in Egypt, Fouad et al., in August 2021, reported 12 cases with rhino-orbital-cerebral mucormycosis presenting to a university hospital in Cairo during the first viral pandemic wave including 6 patients with prior or concurrent COVID-19. El-Kholy et al. in 2021 had also conducted a prospective longitudinal study on invasive fungal sinusitis that spanned the duration of the second Egyptian pandemic wave at a university hospital in another governorate, Mansoura, and detected 28 cases with CO-MUCOR. Alfishawy et al., in 2021, reported 21 patients with CO-MUCOR from 11 different hospitals in Metropolitan Cairo during the third pandemic wave, of which 19 (90.5%) had DM and all had received corticosteroid treatment [18, 19].

The previous findings of our study and previous studies lead us to the conclusion that Egypt comes second to India in the number of published reports of CO-MUCOR cases. The high prevalence of DM in the country, which ranges in recent cluster analyses in different regions from 16.7% (Asaad et al., 2018) to 20.9% (AlSawahli et al., 2019), could explain the burden of CO-MUCOR. Other factors noticed in our sample that can contribute to the disease load include delay in presentation (median 30 days), inadvertent use of corticosteroids in mild-moderate cases (88% of patients), and self-medication for viral infection at home instead of seeking hospital care for moderate-severe cases [20, 21].

The high surge of CO-MUCOR in diabetics and those receiving steroids can be contributed to the modulation of immune system. Neutrophils and macrophages kill sporangiospores and hyphal forms of *Mucorales* and constitute the main barrier to invasion [22].

An adaptive immune response is stimulated and characterized by strong Th-17 activation that initiates a stronger neutrophil response. Simultaneously, the immune response to COVID-19 is complex. Neutrophils in the nasopharyngeal epithelium demonstrate markers of premature activation, while adaptive immunity cells (T cells, NK cells, and B cells) are reduced in numbers [23].

These abnormalities tend to peak in the 2nd week, which may explain the clustering of CO-MUCOR cases in the 3rd week after the appearance of the COVID-19 symptoms. Furthermore, endothelial dysfunction and vasculopathy due to COVID-19 can support angioinvasion and the spread of mucorales [24, 25].

Diabetes or impaired glucose tolerance is reported in the majority of patients with mucormycosis with or without COVID-19. Hyperglycemia inhibits neutrophil chemotaxis, macrophage phagocystosis, and degranulation of NK cells [25]. Promote the expression of surface glucose-regulated protein (GRP78) on the endothelium, which is essential for mucorales invasion. Additionally, supraphysiological stress during COVID-19 illness and viral-mediated islet cell damage can contribute to hyperglycemia. Prolonged corticosteroid intake is a risk factor for the development of mucormycosis. This may be mediated by inhibition of macrophages and neutrophils and the tendency to cause hyperglycemia [26].

We noticed the predominance of headache and facial pain in most patients (69.7%) with CO-MUCOR which is consistent with Desai et al. (2021) who studied 100 patients with a documented history of SARS-CoV-2 infection; the most common complaints were headache and facial pain (55%). Also, Garg et al. (2021) reported a series of 10 cases of CO-MUCOR, and all of them (100%) complained of headache. The difference in percentage may be attributed to smaller sample size in their study [27, 28].

On examination, ophthalmoplegia was a common sign and was present in more than half of patients (54.5%), which is in line with Abdelsamie et al. in 2022 that reported the same percent (54.5%), and also was consistent with El-Kholy et al., in 2021, Desai et al. in 2021, and Mitra et al. in 2021 who found restricted eye movements were present in 63.9%, 58%, and 41% of patients, respectively [27, 29, 30].

In our study, 57.6% of CO-MUCOR patients had unilateral sinus affection. This finding disagrees with Abdelsamie et al. in 2022 who studied 22 patients with CO-MUCOR, and all of them had unilateral sinus affection [29].

In our study, we found that the left ethmoid sinus (81.8%) was the most commonly infected with CO-MUCOR. These finding is consistent with Sharma et al. (2021) study that reported 100% involvement of the ethmoid sinus in all 23 patients included. On the other hand, Nagalli and Kikkeri in their systematic review of the literature in 2021 found that the maxillary sinus was most commonly infected (47.4%) [31].

In our study, all patients received systemic antifungal. About two-thirds of the patients (24 patients, 72.7%) underwent endoscopic sinus surgery for mucormycosis. This agrees closely with Abdelsamie et al. in 2022 who reported that 90.9% of the patients received liposomal amphotericin B, and 81.8% of the patients underwent surgical debridement, and the mortality rate was 27.3% [29].

The overall mortality rate in our study was 27.3% (9 out of 33); this is equal to the overall mortality rate reported by Abdelsamie et al. in 2022, 27.3% (6 out of 22 patients). This close agreement may be due to the fact that both studies were performed in Egypt with similar predisposing factors and similar resources of treatment and more or less similar sample size [29].

On the other hand, the overall mortality in the COSMIC report was 14% (Sen et al., in 2022) which is markedly lower than in our patient group (27.3%). This could probably be attributed to the delay in presentation which is reflected by the median onset of CO-MUCOR following COVID-19 symptoms (13 days in the COSMIC report versus 30 days in our group) [9].

ConclusionThe current mucormycosis epidemic was precipitated by a unique confluence of risk factors: diabetes mellitus, widespread use of steroids, and perhaps SARS-CoV-2 infection itself. Restricting steroid use in patients with severe COVID-19 requiring oxygen therapy and screening for and optimally controlling hyperglycemia can prevent COVID–Mucor in a large majority.

Our findings call for the avoidance of steroids in mild COVID-19 in view of the risk of CAM. In general, it appears that neutrophil dysfunction (due to COVID-19, hyperglycemia, and steroids) and endothelial dysfunction (due to diabetes and COVID-19) may be the main pathogenetic mediators of CAM.

## Data Availability

The datasets used during the current study are available from the corresponding author on reasonable request.
